# The 2010s in clinical drug-eluting stent and bioresorbable scaffold research: a Dutch perspective

**DOI:** 10.1007/s12471-020-01442-w

**Published:** 2020-08-11

**Authors:** H. Kawashima, P. Zocca, R. A. Buiten, P. C. Smits, Y. Onuma, J. J. Wykrzykowska, R. J. de Winter, C. von Birgelen, P. W. Serruys

**Affiliations:** 1 Department of Clinical and Experimental Cardiology, Heart Center, Amsterdam Cardiovascular Sciences, Amsterdam UMC, University of Amsterdam, Amsterdam, The Netherlands; 2 Department of Cardiology, National University of Ireland, Galway (NUIG), Galway, Ireland; 3 Department of Cardiology, Thoraxcentrum Twente, Medisch Spectrum Twente, Enschede, The Netherlands; 4 Health Technology and Services Research, Faculty of Behavioural Management and Social Sciences, Technical Medical Centre, University of Twente, Enschede, The Netherlands; 5 Department of Cardiology, Maasstad Hospital, Rotterdam, The Netherlands; 6 Imperial College London, London, UK

**Keywords:** All-comer(s), Bioresorbable scaffold, Drug-eluting stent, Percutaneous coronary intervention, Randomised trial

## Abstract

Dutch researchers were among the first to perform clinical studies in bare metal coronary stents, the use of which was initially limited by a high incidence of in-stent restenosis. This problem was greatly solved by the introduction of drug-eluting stents (DES). Nevertheless, enthusiasm about first-generation DES was subdued by discussions about a higher risk of very-late stent thrombosis and mortality, which stimulated the development, refinement, and rapid adoption of new DES with more biocompatible durable polymer coatings, biodegradable polymer coatings, or no coating at all. In terms of clinical DES research, the 2010s were characterised by numerous large-scale randomised trials in all-comers and patients with minimal exclusion criteria. Bioresorbable scaffolds (BRS) were developed and investigated. The Igaki-Tamai scaffold without drug elution was clinically tested in the Netherlands in 1999, followed by an everolimus-eluting BRS (Absorb) which showed favourable imaging and clinical results. Afterwards, multiple clinical trials comparing Absorb and its metallic counterpart were performed, revealing an increased rate of scaffold thrombosis during follow-up. Based on these studies, the commercialisation of the device was subsequently halted. Novel technologies are being developed to overcome shortcomings of first-generation BRS. In this narrative review, we look back on numerous devices and on the DES and BRS trials reported by Dutch researchers.

## Dutch contribution to the field

- Dutch researchers reported the first cases and registries of late stent thrombosis of first-generation drug-eluting stents (DES).
- Following the first introduction of the concept of all-comer trials in the LEADERS trial, multiple randomised control trials (RCT) were performed by Dutch leading investigators to compare, in all-comer populations, the first- and second-generation DES or between second-generation DES. These trials have demonstrated the improved outcomes of the second-generation compared with first-generation DES and in general, comparable results between second-generation DES.
- In specific populations, such as ST-segment elevation myocardial infarction and chronic total occlusion, RCT were performed to compare the first- and second-generation DES or between second-generation DES.
- Initial favourable imaging results from the first-in-man trials of the first-generation Absorb led to the wide adoption of the technology of the bioresorbable scaffold. However, the randomised ABSORB II and AIDA trials demonstrated a higher scaffold thrombosis rate and subsequently, the device was withdrawn from the market.

## Introduction

The introduction of bare metal stents (BMS) broadened the options for treating patients with obstructive coronary artery disease, which in the mid-1980s consisted of medical therapy, balloon angioplasty, or bypass surgery [1]. Dutch investigators were among the first to assess the possibilities and limitations of coronary stents [2–4]. A first multicentre study identified early thrombotic stent occlusion as the most important limitation, and it also revealed the problems of restenosis and late stent occlusion [5]. In the 1990s, research went on to refine stents, implantation strategies, and concomitant pharmacological therapy. The BENESTENT‑I and II trials played a pivotal role in establishing coronary stenting as a valuable therapy [6, 7]. Lessons from intravascular ultrasound [8] resulted in the use of larger balloon sizes and higher balloon pressures, which optimised stent expansion and apposition, made it possible to simplify the concomitant pharmacological treatment, and profoundly reduced the incidence of stent thrombosis. For many years in-stent re-stenosis was the main limitation of stenting, before the favourable outcome of the durable polymer-coated Cypher sirolimus-eluting stent (SES) in the RAVEL study led to a rapid adoption of the first-generation drug-eluting stents (DES) [9]. Then the early enthusiasm about DES was subdued by discussions about an increased risk of very-late stent thrombosis and mortality [10, 11]. These discussions stimulated the development and rapid implementation of newer-generation DES with refined, more biocompatible coatings during the 2010s. DES research in the 2010s was characterised by numerous large-scale randomised trials in all-comers and patients with minimal exclusion criteria. In addition, bioresorbable scaffolds (BRS) were designed to provide temporary scaffolding and then to disappear later with biodegradation of the device. Igaki-Tamai, the first BRS without drug elution, was implanted in a human in 1999 [12] and demonstrated restenosis rates similar to BMS. In the 2000s, the first and second iteration of an everolimus-eluting BRS were evaluated in the first-in-man studies ABSORB Cohort A and B [13, 14]. The favourable imaging and clinical results of these studies generated enthusiasm for BRS technology. However, the company decided on a worldwide halt to sales of the first-generation Absorb in September 2017 due to a higher rate of major adverse cardiovascular events (MACE) and scaffold thrombosis (ScT), documented in the randomised ABSORB II, III, China, Japan, and AIDA trials [15–18]. To overcome shortcomings of first-generation BRS, new-generation BRS are being developed to improve acute and long-term safety and efficacy.

## Scope of this review

In the present narrative review, focused on major randomised DES and BRS trials, we briefly look back on numerous studies that Dutch researchers reported during this decade. In January 2020, we searched on PubMed.gov for clinical studies written in English, published from 1 January 2010 to 31 December 2019, containing the terms “coronary”, and “stent” or “scaffold”, and “randomised” in the manuscript title or abstract, containing an abstract, and containing “Netherlands” in the affiliations. We reviewed the summary pages of these manuscripts, searching for first or senior corresponding authors (or principal investigators) affiliated to Dutch medical centres or clinical research organisations. Published abstracts and unpublished oral presentations were not considered. While we aimed at comprehensiveness of major randomised DES and BRS trials reported by Dutch researchers, we cannot exclude that an important study regrettably may not be mentioned.

## Assessment of durable polymer DES in myocardial infarction

In the setting of ST-segment elevation myocardial infarction (STEMI), there was little clinical evidence in favour of using DES and even stents in general [19, 20]. Therefore, several randomised trials compared DES versus BMS in this setting. Consecutively, newer-generation DES were compared with first-generation DES. Two trials compared the first-generation Cypher SES with BMS in STEMI patients and reported long-term follow-up data. The DEBATER trial assessed 907 patients and found, at 1 year, that the SES reduced the primary endpoint of major adverse cardiac and cerebrovascular events (16.5% vs. 25.8%, *p* = 0.001), mainly driven by less repeat revascularisations [21]. After 5 years, the primary endpoint was no longer significantly different (*p* = 0.12), and the very-late (1–5 years) stent thrombosis rates were 2.0% versus 0.7% (*p* = 0.12) [22]. After 5 years, the MISSION! Intervention study also observed a trend towards more very-late (>1 year) stent thromboses in SES (4.1% vs. 0.7%, *p* = 0.07) [23]. Two DES trials assessed first-generation Taxus paclitaxel-eluting stents (PES) versus BMS and reported 5‑year follow-up [24, 25]. In the PASSION trial, 619 STEMI patients showed no significant between-stent difference in MACE, and there was no significant difference in definite-or-probable stent thrombosis at 5 years [24]. The EXAMINATION trial randomised 1498 STEMI patients to PES or BMS, and after 5 years the rates of the main composite clinical endpoint and of stent thrombosis were comparable for both stents [25]. Notably, in both trials very-late stent thrombosis was seen almost exclusively in PES [24, 25].

The newer-generation durable polymer Xience everolimus-eluting stent (EES) was assessed in the XAMI trial, which randomised 625 patients with acute myocardial infarction (MI) to Xience EES or Cypher SES [26]. At 1‑year, the rate of MACE was lower in EES (4.0% vs. 7.7%, *p* = 0.048), and the definite-or-probable stent thrombosis rates were 1.2 and 2.7% (*p* = 0.21) [26]. After 3 years, event rates remained low and similar [27]. The APPENDIX-AMI trial randomised 977 all-comer patients to receive EES or SES, including 112 patients with STEMI, and showed similar 2‑year rates of the primary composite endpoint and of stent thrombosis [28]. In a pooled analysis of all patients from XAMI and APPENDIX-AMI, both DES showed similar 2‑year outcomes. Moreover, findings were similar in the large subpopulation of STEMI patients [29].

## Evaluation of self-expanding DES in myocardial infarction

In the setting of acute MI, true vessel size is sometimes underestimated and stents with self-expanding properties may provide theoretical advantages. The APPOSITION II study randomised 80 patients to receive the self-expanding STENTYS SES or BMS. Optical coherence tomography 3 days after stenting showed a lower rate of malapposed stent struts in STENTYS (0.6% vs. 5.5%, *p* < 0.001), but clinical outcomes were similar at 6‑month follow-up [30]. The APPOSITION IV trial randomised 152 STEMI patients to receive STENTYS SES or Resolute zotarolimus-eluting stents (ZES) and showed with optical coherence tomography that late stent strut apposition and strut coverage were similar for both devices at 9 months, but achieved earlier in patients treated with STENTYS [31]. A single-centre experience in 120 patients, treated with STENTYS in highly complex coronary lesions and atypical coronary anatomies, revealed reasonable clinical results at 5 years [32].

## Assessment of newer-generation DES in patients with low-to-moderate complexity

The randomised SPIRIT II and III trials compared Xience EES with first-generation Taxus PES in de-novo lesions. At 2 years, a patient-level pooled analysis in data from 1302 patients found the MACE rate to be significantly lower in EES (7.1% vs. 12.3%, *p* = 0.0014) while there was no significant difference in stent thrombosis [33]. At 5‑year follow-up of the SPIRIT II trial, which randomised 300 patients to EES or PES, cardiac mortality (1.5% vs. 7.3%, *p* = 0.015) and ischaemia-driven MACE (8.0% vs. 18.1%, *p* = 0.018) rates were significantly lower in EES, and the stent thrombosis rate was numerically lower in EES (0.9% vs. 2.8%) [34]. A subgroup analysis of the EES group at 3 years showed that treatment of calcified lesions was safe and associated with quite favourable repeat revascularisation rates [35].

Several other newer-generation DES were assessed in small-sized randomised trials with angiographic primary endpoints. The EXCELLA II trial randomised 210 patients to treatment with DESyne novolimus-eluting stents or Endeavor ZES. At 9‑month angiographic follow-up, it showed superiority of the DESyne stent in late lumen loss (*p* < 0.0001) [36]. After 5 years, patients treated with DESyne showed a significantly lower incidence of device (7.9% vs. 19.7%, *p* = 0.022) and patient-oriented events (23.7% vs. 40.8%, *p* = 0.016) [37]. In the PIONEER II trial, 170 patients were allocated to implantation of a biodegradable polymer-coated BuMA Supreme SES or Resolute ZES. After 9 months, the angiographic primary non-inferiority endpoint of in-stent late lumen loss was not met, but this result did not translate into impairment in 12-month clinical outcome [38]. The NIREUS trial assessed the BioNIR ridaforolimus-eluting stent and randomised 302 patients to treatment with this device or Resolute Integrity ZES. At 6‑month angiographic follow-up, the rates of late lumen loss were similar for both DES (*p* = 0.79) [39].

## All-comers trials to assess new durable polymers DES

In the 2010s, several large-scale randomised trials applied only few exclusion criteria in order to assess newer-generation DES in patient populations that resembled routine clinical practice. The single-centre COMPARE trial assigned 1800 all-comer patients to treatment with Xience EES or Taxus PES and found the 1‑year primary composite clinical endpoint to be significantly lower in EES (6.2% vs. 9.1%, *p* = 0.02) [40]. In addition, in EES the event rates were lower for stent thrombosis (0.7% vs. 2.5%, *p* = 0.002), MI, and target vessel revascularisation [32]. At 5‑year follow-up, the early superiority in safety and efficacy of EES over PES was sustained with main endpoint rates of 18.4% versus 25.1% (*p* = 0.0005) and definite-or-probable stent thrombosis rates of 3.1% versus 5.9% (*p* = 0.005) [41].

Other trials compared two newer-generation DES with each other. In the multicentre Resolute All Comers trial, 2292 all-comer patients were randomised to treatment with Resolute ZES or Xience V EES to compare the device-oriented primary composite clinical endpoint at 1 year [42]. The rates of this endpoint were similar (8.2% vs. 8.3%, *p* = 0.92), and there were also no significant between-group differences in secondary endpoints [42]. In patients with bifurcated target lesions, treatment with these DES showed favourable results [43]. At the final 5‑year follow-up, similar safety and efficacy outcomes were sustained between the two DES [44].

The TWENTE trial assigned 1391 patients (all-comers, except for STEMI) to treatment with Resolute ZES or Xience V EES and showed, after 1 year, a similar incidence of the primary endpoint target vessel failure (8.2% vs. 8.1%, *p* = 0.94) [45]. The single-centre trial enrolled 81.4% of all 1709 eligible patients while the non-enrolled eligible patients were also treated (in a non-randomised fashion) with ZES or EES, showing 1‑year outcomes similar to the TWENTE trial participants [46]. After 5 years, TWENTE trial participants showed low and similar stent thrombosis rates for ZES and EES (1.0% vs. 0.6, *p* = 0.37) [47]. Long-term outcomes from the non-enrolled patients supported the validity of these findings [47].

Then, durable polymer DES with more flexible stent designs and/or improved radiographic visibility became available. The DUTCH PEERS (TWENTE II) trial compared the cobalt-chromium Resolute Integrity ZES and the platinum-chromium Promus Element EES in 1811 all-comers, treated at four Dutch centres. The 1‑year rate of the primary endpoint target vessel failure was similar for both DES (6.1% vs. 5.2%, *p* = 0.42). Secondary endpoints showed no significant between-DES difference, and longitudinal deformation was only seen in a few patients treated with the angiographically more visible Promus Element EES (1.0% vs. 0%, *p* = 0.002); however, this was not associated with adverse events [48]. Subgroup analyses in patients treated for bifurcation lesions and for MI [49, 50], and the trial’s 5‑year follow-up underlined the favourable findings of these two DES [51].

The BIONYX (TWENTE IV) trial investigated the next iteration of the ZES (Resolute Onyx) that uses a newly designed stent, made from a novel swaged shape composite wire and has an outer cobalt-chromium layer with a dense platinum-iridium core, which improves X‑ray visibility and allows to slightly reduce strut thickness [52]. The trial randomised 2516 all-comers at 7 centres to treatment with Resolute Onyx ZES versus Orsiro SES and showed, at 1‑year follow-up, low and similar rates of the primary endpoint target vessel failure (4.5% vs. 4.7%, *p* = 0.77%). In addition, the rates of definite-or-probable stent thrombosis were low (0.1% vs. 0.7%, *p* = 0.011) [52].

## All-comers trials to assess biodegradable polymer DES

DES that leave behind only a bare metal stent after polymer resorption were developed, as theoretically they might improve long-term outcome after stenting. The LEADERS, GLOBAL LEADERS, and COMPARE multicentre trials investigated two types of early-generation biodegradable polymer-coated biolimus A9-eluting stents (BES) with thick stent struts. The LEADERS trial, which randomised 1707 all-comers to treatment with BioMatrix BES versus Cypher SES, had established non-inferiority of the BES at 9 months. At 5‑year follow-up, the rate of the main composite clinical endpoint did not differ between DES groups (22.3% vs. 26.1%, *p* = 0.83). But the very-late definite stent thrombosis rate was significantly lower in BES (0.7% vs. 2.5%, *p* = 0.003), which corresponded with a significant reduction in thrombosis-associated clinical events [53]. For BES, subgroup analyses at 5‑year follow-up suggested a superior long-term efficacy in bifurcated target lesions [54] and better clinical outcomes in MI, especially in patients with STEMI [55]. The GLOBAL LEADERS trial was designed to compare two different antiplatelet regimes in 15,968 patients who were treated with the BioMatrix BES. At 2‑year follow-up, the rate of the primary composite endpoint of all-cause mortality and non-fatal MI was low in all patients [56]. In the COMPARE II trial, 2707 patients were assigned to treatment with Nobori BES or durable polymer EES (Xience V, Xience Prime, or Promus). At 1‑year follow-up, there was no significant difference in the incidence of the primary composite clinical endpoint which occurred in 5.2% versus 4.8% [57]. Long-term follow-up at 5 years showed that treatment with BES versus EES resulted in main clinical endpoint rates of 15.2% versus 12.9% (*p* = 0.12) and similar combined safety and combined efficacy endpoints. Furthermore, the 5‑year definite stent thrombosis rate showed no significant difference between DES groups (1.5% vs. 0.9%; *p* = 0.17) [58]. In patients with diabetes, 5‑year event rates were also similar for both DES [59].

Subsequently, biodegradable polymer-coated DES with very thin stent struts were developed. The multicentre BIO-RESORT (TWENTE III) trial randomised 3514 all-comer patients (70% had acute coronary syndromes) to treatment with Synergy EES or Orsiro SES versus durable polymer Resolute Integrity ZES to assess the primary endpoint target failure after 1 year. This endpoint was met in EES by 4.7% and in SES by 4.7% versus 5.4% in ZES (*p* = 0.45 and *p* = 0.46, respectively). Secondary endpoint rates were also low and similar [60]. At 3‑year follow-up, all DES showed similar and favourable medium-term safety and efficacy, and the definite-or-probable stent thrombosis rates were low (1.1, 1.1, and 0.9%) [61]. A subgroup analysis in patients treated in small coronary vessels suggested that the risk of repeated revascularisations may be independently reduced by use of the Orsiro SES (vs. Resolute Integrity ZES, *p* = 0.02) [62].

Meanwhile, other biodegradable polymer-coated DES with very thin stent struts have been developed that also elute sirolimus from their biodegradable polymer coatings. In the multicentre DESSOLVE III trial, 1398 all-comer patients were randomised to implantation of biodegradable polymer MiStent SES or Xience EES. At 1‑year follow-up, the device-oriented primary composite clinical endpoint occurred in 5.8% versus 6.5%, and there was no significant between-DES difference in secondary endpoints [63]. The TALENT trial assessed 1435 all-comer patients at multiple centres, who were assigned to the implantation of biodegradable polymer Supraflex SES or Xience EES. After 1 year, the device-oriented primary composite clinical endpoint was similar in SES and EES (4.9% vs. 5.3%). The incidence of secondary endpoints was low and similar for both devices [64].

## All-comers trial to assess new polymer-free DES

The polymer-free amphilimus-eluting stent (Pf-AES) releases the antiproliferative drug by means of an amphiphilic carrier, stored in abluminal laser-dug wells [65]. The ReCre8 study randomised a total of 1502 all-comers at three centres to treatment with Pf-AES or Resolute Integrity ZES. The primary endpoint occurred in 6.2% versus 5.6% (*p* = 0.67), and there was no difference in secondary endpoints [65].

## Registries assessing endothelial progenitor cell-capturing DES

Encouraging findings with the Genous endothelial progenitor cell-capturing stent [66–68] resulted in the development of the COMBO stent, which elutes sirolimus from a biodegradable polymer on the abluminal side of the strut and an anti-CD34+ antibody layer on the luminal side to attract circulating endothelial progenitor cells [69]. The REMEDEE registry [70] as well as the COMBO Collaboration [69], which is a patient-level pooled analysis of 3614 patients from the multicentre registries MASCOT and REMEDEE, have shown favourable clinical outcomes after 1 year [69, 70].

## Trials assessing DES in chronic totally occluded coronary arteries

The PRISON series of randomised clinical trials assessed various coronary DES for treating chronic total occlusion lesions. The PRISON II trial randomised 200 patients to treatment with Cypher SES or BMS and found lower 5‑year target vessel revascularisation and MACE rates in SES (*p* = 0.009 and *p* < 0.001) [71]. Furthermore, there was a trend towards more definite stent thromboses in SES. In PRISON III, 304 patients were randomised to receive Cypher SES or ZES (31% Endeavor, 69% Resolute) [72]. The primary angiographic endpoint was in-segment late lumen loss after 8 months, which was lower in SES versus Endeavor ZES (*p* = 0.0002) but not in SES versus Resolute (*p* = 0.58). Three-year follow-up showed a relatively low incidence of adverse events [73]. The PRISON IV trial compared Orsiro SES and Xience EES in 330 patients [74]. The primary non-inferiority endpoint of in-segment late lumen loss at 8 months was not met for the SES vs. EES, and the incidence of binary angiographic restenosis was significantly higher with SES while the stent thrombosis rate was similar [74]. A secondary analysis of this trial suggested that the inferior performance of the SES was pronounced when smaller SES (≤3 mm diameter) were used, which have struts with a thickness of 60 μm [75].

## Trials assessing Absorb BRS

The everolimus-eluting Absorb scaffold consists of a semi-crystalline poly-L-lactic acid backbone coated with poly‑D, L‑lactide acid with a strut thickness of 157 μm. The first-in-man trial (ABSORB Cohort A, 30 patients) of the first-generation Absorb demonstrated that after bioresorption of the scaffold, treated coronary arteries had their vasomotion function restored with possible late lumen enlargement [13]. At 5‑year follow-up, MACE rate was 3.4% and no ScT was observed [76]. Using the second iteration of the Absorb, the ABSORB Cohort B (101 patients) demonstrated a 5-year MACE rate of 11.0% without any ScT [14]. These results generated enthusiasm for the technology of BRS with the perspective of avoiding the late consequence of a permanent foreign body in the coronary artery.

In the ABSORB II trial, 501 patients with de novo coronary lesions were randomised in a 2:1 ratio to treatment with Absorb or Xience EES [15]. The trial did not show superiority for Absorb with regards to the primary endpoint vasomotor reactivity at 3‑year follow-up. In addition, the trial did not meet its co-primary endpoint of non-inferior late luminal loss for the Absorb compared with the EES, which was found to have a significantly lower late luminal loss than the Absorb BRS [77]. In a sub-study, late lumen enlargement and expansive remodelling between the Absorb and the EES were compared [78]. At 3‑year follow-up, the relative change in mean vessel area was significantly greater with Absorb compared with the EES (6.7 ± 12.6% vs. 2.9 ± 11.5%, *p* = 0.003); the relative change in mean lumen area was significantly different between the two arms (1.4 ± 19.1% vs. −1.9 ± 10.5%, *p* = 0.031). Definite or probable ScT was more frequently observed in the Absorb group than in the EES group (3.0% vs. 0.0%, *p* = 0.033) [79]. However, between 3 and 5 years, no additional ScT was observed.

In the all-comer AIDA trial [18, 80], 1845 patients undergoing percutaneous coronary intervention were randomised to receive either the Absorb or Xience EES. There was no significant difference in the rate of the primary endpoint target vessel failure. Definite or probable ScT up to 2 years occurred in 31 patients in the Absorb group as compared with 8 patients in the EES group (3.5% vs. 0.9%, *p* < 0.001).

In the ABSORB-STEMI TROFI II trial, 191 patients with STEMI were randomly allocated 1:1 to treatment with the Absorb or Xience Xpedition EES [81]. This trial demonstrated that implantation of culprit lesions with the Absorb in the setting of STEMI resulted in a nearly complete arterial healing which was comparable with the healing of EES at 6 months.

In the VANISH trial, 60 patients were randomised to treatment with Absorb versus Xience Prime EES and positron emission tomography perfusion imaging was used to assess the effects of both treatments on (hyperaemic) myocardial blood flow and coronary flow reserve over 3‑year period [82]. At 1 month, no differences in absolute myocardial perfusion were observed between the two groups. Coronary flow reserve of the treated myocardial territory was significantly lower in patients treated with the Absorb (3.09 ± 0.94 vs. 3.57 ± 0.85, *p* < 0.05).

In a patient-level meta-analysis of 3389 patients (2164 treated with Absorb vs. 1225 treated with Xience EES) from the randomised trials with patients with chronic coronary syndromes [16, 17, 77, 79, 83, 84], the target lesion failure and ScT rates with the Absorb were significantly higher from 0–3 years compared with the Xience EES (14.9% vs. 11.6%, *p* = 0.03, and 2.5% vs. 0.8%, *p* = 0.002, respectively) [85]. However, from 3 to 5 years, the event rates were no longer different between the two devices, and numerically fewer ScT occurred with the Absorb after 3 years. These data imply that the risk period for the Absorb ends at 3 years, suggesting that if improved scaffolds and technique were able to overcome these early risks, the benefits of ‘leaving nothing behind’ might be realised.

The COMPARE ABSORB trial was a prospective, single-blind multicentre trial performed in 45 European sites. The trial was designed to enrol 2100 patients randomised in 1:1 ratio to Absorb or Xience EES [86]. Specific advice on implantation technique including mandatory pre-dilation, sizing, and post-dilation (PSP) was used in the Absorb arm. This trial started with such initial experiences and was one of the first trials with Absorb in which both a specific PSP implantation technique was implemented in the protocol from the start, and that included only experienced operators/centres for enrolment. However, the trial was stopped prematurely by the Data and Safety Monitoring Board in August 2017 based on the results observed in the randomised Absorb trials (ABSORB II, III, China, Japan, and AIDA).

## Other BRS and future development

Although the first-generation BRS was abandoned in clinical practice based on the outcomes of randomised clinical trials, better outcomes may be obtained with new-generation BRS [87]. The imaging analysis of the Absorb suggested that very-late ScT could be attributed to the intra-luminal dismantling caused by the insufficient tissue encapsulation of the strut at long-term follow-up, especially at the period of decreasing mechanical property due to bioresorption [88]. Therefore, new-generation BRS should facilitate the early tissue coverage and late tissue encapsulation of the scaffold strut prior to complete bioresorption of the device. Early embedment and encapsulation could be promoted by a low profile of thin struts with small footprints. Increases in tensile stress of the bioresorbable material with reinforced mechanical properties are an indispensable improvement to achieve that goal. In general, new-generation BRS uses bioresorbable materials with enhanced mechanical properties. ‘STENTiT’ has a unique technological approach to manufacture by electrospinning polymeric tube with microfiber. In a rat model, the implantation of the STENTiT scaffold resulted in the formation of an elastic layer in the endoluminal border: the histomorphological appearance mimics the structure of the internal mammary artery. The other Dutch-based company ‘Xeltis’ is developing bioresorbable devices using supra molecule [89].

## Conclusion

This review has shed a light on the Dutch contribution to clinical DES and BRS research during the 2010s. During this decade, DES were refined, thoroughly investigated, and rapidly adopted in routine clinical practice. DES research was characterised by a sizeable series of large-scale randomised ‘all-comers’ trials and in patients with minimal exclusion criteria. Most new DES used more biocompatible durable polymer or biodegradable polymer coatings, and there was a trend toward more flexible stent designs and thinner stent struts. During the 2010s, there was a steady decrease in adverse clinical event rates following DES implantation that does not only reflect advances in procedural strategy and concomitant pharmacological treatment, but also refinements in DES technology that were clinically assessed in the trials that we have discussed in this review. The concept of BRS—‘a device that disappears after it has done its job’—is attractive from the viewpoint of the patient. Since the 1990s with the advent of the everolimus-eluting BRS, multiple randomised clinical trials have been performed. However, so far, the device has failed to demonstrate a clinical benefit over the conventional metallic stent. Further development of the bioresorbable technology is expected to overcome the shortcoming of first-generation BRS.
